# Ischemic lesions to the inferior frontal cortex slow perceptual switching

**DOI:** 10.1016/j.isci.2026.114943

**Published:** 2026-02-07

**Authors:** Merve Fritsch, Jochen Michely, Lucca Jaeckel, Ida Rangus, Christoph Riegler, Jan F. Scheitz, Christian Nolte, Philipp Sterzer, Veith Weilnhammer

**Affiliations:** 1 Department of Psychiatry and Neurosciences, Charité - Universitätsmedizin Berlin, Berlin, Germany; 2 Helen Wills Neuroscience Institute, University of California, California, Berkeley, CA, USA; 3 Berlin Institute of Health (BIH) at Charité - Universitätsmedizin Berlin, Berlin, Germany; 4 Department of Psychiatry (UPK), University of Basel, Basel, Switzerland; 5 Department of Neurology with Experimental Neurology, Charité - Universitätsmedizin Berlin, Berlin, Germany; 6 Center for Stroke Research Berlin (CSB), Charité - Universitätsmedizin Berlin, Berlin, Germany; 7 Center for the Study of Aphasia Recovery, University of South Carolina, Columbia, SC, USA; 8 Max Planck UCL Centre for Computational Psychiatry and Ageing Research, London, UK

**Keywords:** Imaging anatomy, Radiology, Cognitive neuroscience

## Abstract

In bistable perception, conscious experience alternates between two interpretations of an ambiguous stimulus. Functional imaging shows the activation of the inferior frontal cortex (IFC) during such reversals. However, it has remained unclear whether IFC involvement reflects post-perceptual processes such as attention or motor behavior, or contributes to perceptual updating itself. We examined bistable perception in patients with chronic ischemic stroke lesions. Patients with right middle cerebral artery (rMCA) lesions affecting the IFC (*n* = 8) exhibited fewer spontaneous perceptual changes than those with rMCA lesions sparing the IFC (*n* = 13), after controlling for age, sex, stroke burden, and non-perceptual task performance. Group differences were observed in a small sample with heterogeneity in lesion characteristics, including lesion volume and severity, which limit definitive causal inference. These preliminary results indicate that IFC damage slows updating of conscious experience in response to ambiguous sensory input, supporting a contributory role of the prefrontal cortex in shaping conscious perception.

## Highlights

• Ischemic lesions involving the inferior frontal cortex alter bistable perceptual dynamics
• Patients with IFC lesions show reduced rates of perceptual switching after stroke
• Effects persist after controlling for lesion volume and clinical stroke severity
• Findings support a role of the IFC in updating perception under sensory ambiguity

## Graphical abstract

**Figure:**
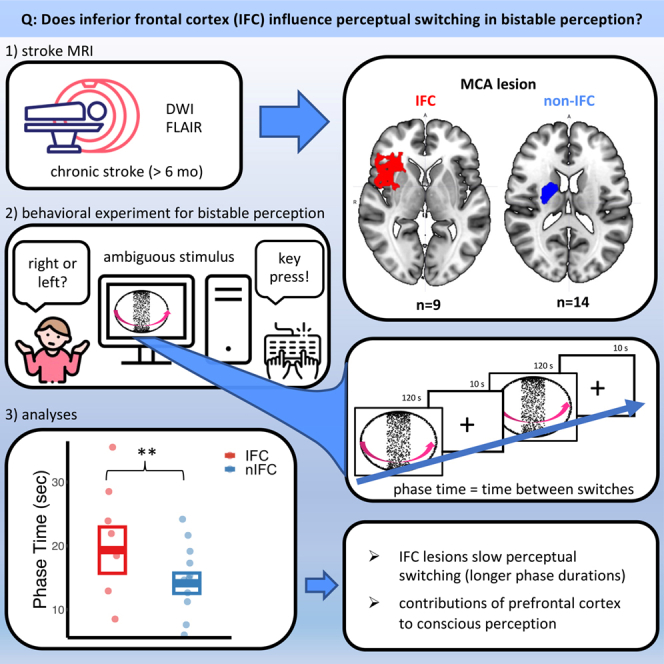

## Introduction

The signals registered by our senses often support multiple and conflicting interpretations. Conscious experience, however, portrays only one view of the world at a time.^1^ This discrepancy becomes evident during bistable perception, where ambiguous stimuli contain equal sensory information for two mutually exclusive percepts, leading to spontaneous changes between two conscious experiences.^2^^,^^3^

The inferior frontal cortex (IFC) has been shown to activate at the time of changes in conscious experience during bistable perception. Some suggest that IFC activity reflects cognitive processes that occur in the wake of conscious experiences, supporting non-perceptual functions such as task engagement, motor behavior, or introspection.^4^^,^^5^ Others argue that the IFC plays a causal role in transforming ambiguous sensory signals into unambiguous conscious experiences,^6^^,^^7^ either by representing their content^8^^,^^9^ or by triggering changes between conflicting interpretations of ambiguous stimuli.^10^^,^^11^

This study investigates how loss-of-function in patients with chronic (≥6 months) ischemic stroke lesions in IFC impacts the resolution of perceptual conflict in bistable perception. To this end, 23 patients (9 with MCA-lesions involving IFC and 14 with MCA-lesions excluding IFC) participated in a bistable perception task and reported perceived perceptual changes while viewing an ambiguous stimulus.

We hypothesized that patients with stroke with right middle cerebral artery (MCA) lesions, including the IFC, would experience fewer spontaneous changes than patients with right MCA lesions excluding IFC.

## Results

### Experimental design and patient characteristics

**Figure 1:**
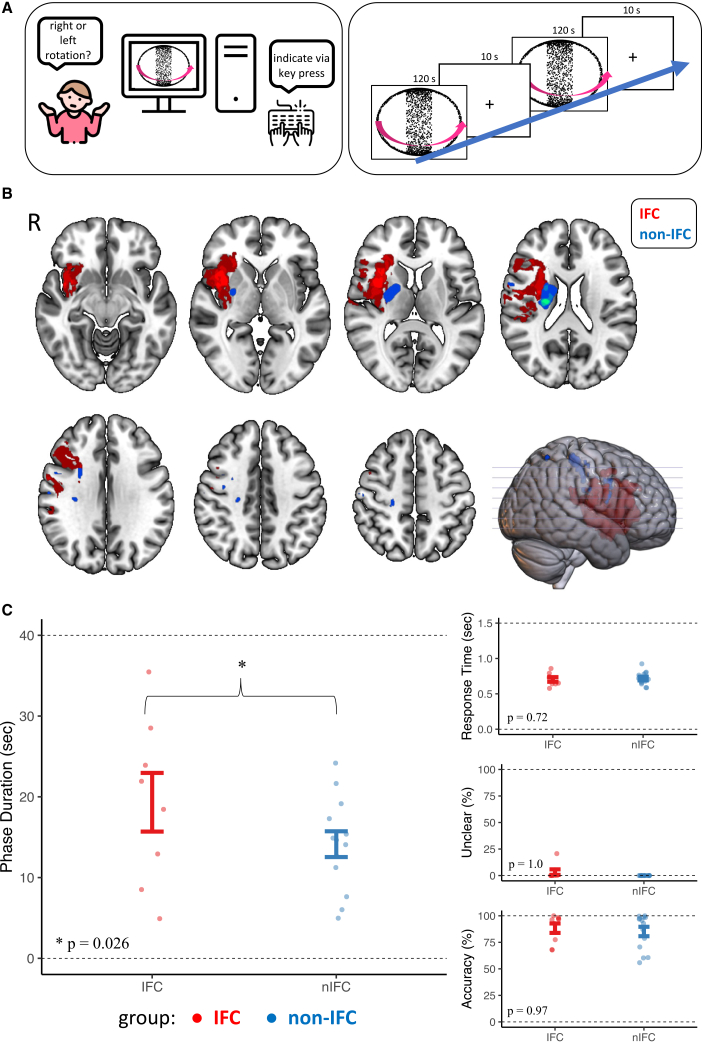
Experimental design, lesion distribution, and behavioral effects of right IFC lesions during bistable perception (A) Experimental Design: (left) - patients underwent a computer-based behavioral task, reporting perceived rotation of a spherical RDK via key press. (right) - Patients performed 4 runs, of which the first showed a disambiguated version of the stimulus. Three runs were completely ambiguous, thus inducing bistable perception. Each run consisted of 6 blocks at 120 s each that were separated by 10 s of fixation intervals. Perceived rotation was reported via button press on a keyboard. (B) Lesion locations of both groups are shown as overlay maps. All patients had lesions in the right MCA territory. Nine participants had lesions involving the right IFC – shown in red. IFC was defined as being located in the area comprised of the anterior insula and the inferior frontal gyrus (pars triangularis and pars opercularis) using the AAL atlas. 14 patients had lesions without the involvement of the right IFC and served as the control group - shown in blue. R = right hemisphere. (C) Results of the behavioral task: (left) Patients with IFC lesions showed significantly longer phase durations between perceptual changes. (right top to bottom) The control parameters “Response Time,” “Unclear Perceptual States,” and “Accuracy” did not differ significantly between groups. Data points represent individual participants. Error bars indicate mean ± SEM. *p* values are shown in the figure; ∗*p* < 0.05.

We recruited 23 patients with chronic ischemic stroke in the right middle cerebral artery (MCA) territory (mean time since stroke onset 16.2 months). Nine patients had MCA lesions affecting the right IFC (“IFC,” 4 female, age 69 ± 9.6 years) and 14 patients had lesions sparing the right IFC (“non-IFC,” 7 female, age 71 ± 11.3 years) (Figures 1B and S2; Table 1 and STAR Methods Section for further information regarding patient characteristics and lesion distribution). Patients participated in a behavioral experiment using a previously established bistable perception task (Figure 1A).^10^ They viewed ambiguous (3 runs) and unambiguous (1 run) versions of a random-dot-kinematogram (RDK), inducing the experience of a rotating sphere, and reported changes in the perceived rotation direction (“right” = front surface toward the right, “left” = front surface toward the left or “unclear”) via key presses on a standard USB keyboard (Figure 1A and Video S1). During the unambiguous run, the rotation direction of a disambiguated RDK changed in random intervals (10 s on average) (Video S2). During the ambiguous runs, participants perceived changes in conscious experience in the absence of any change in the physical stimulus (bistable perception). As shown in previous studies, perceived changes in perception occur almost exclusively at the overlapping configuration in this condition (Figures 1A and S1A and STAR Methods for details).^10^^,^^11^^,^^12^

**Table 1:** Clinical characteristics, behavioral measures, and control parameters of patients with and without right IFC lesions

	IFC	Non-IFC	p
**Clinical data**			

N	9	14	–
Age (y)	69 ± 9.6	71 ± 11.3	*p* = 0.07
Sex (f, %)	44 ± 0.5	46 ± 0.5	*p* = 9.3 x 10^−1^
NIHSS at discharge	2.0 ± 2.6	1.0 ± 1.3	*p* = 9.4 x 10^−9^
Lesion volume (mm^3^)	45154 ± 51694	903.3 ± 1137.3	*p* = 2 x 10^−16^

**Measures of interest**			

Phase Duration (s)	19.33 ± 3.63	14.14 ± 1.6	*p* = 0.026

**Control parameters**			

Response Times (s)	0.69 ± 0.18	0.70 ± 0.16	*p* = 0.72
Accuracy (%)	88.33 ± 13.05	85.0 ± 17.46	*p* = 0.97
Unclear (%)	2.8 ± 16.3	0.95 ± 8.2	*p* = 1

Means and standard deviations of clinical data, measures of interest and control parameters for both groups, respectively. Phase duration was calculated in participants who reported at least one perceptual switch in the disambiguated condition (IFC *n* = 8; Non-IFC *n* = 13).

Video S1. Ambiguous structure-from-motion stimulus inducing bistable perception, related to Figure 1A Ambiguous structure from motion.

Video S2. Disambiguated structure-from-motion stimulus used in the control condition Disambiguated version of the stimulus (during the experiment viewed with red and blue filter glasses).

### Lesions of the right inferior frontal cortex are associated with longer phase durations during bistable perception

To assess whether the IFC has a contributing role in bistable perception, we tested whether phase duration - the elapsed time between perceptual changes in seconds – during the ambiguous runs was longer in patients with structural lesions in the IFC as compared to patients with structural lesions outside of the IFC. In line with our hypothesis, we observed significantly longer phase durations in patients with IFC lesions compared to patients with lesions outside IFC (IFC 19.33 ± 3.63 s, non-IFC 14.14 ± 1.6 s, see Figure 1C) while controlling for age, sex, clinical stroke severity as measured by NIHSS at discharge and lesion volume (T(5.97) = 2.95, *p* = 0.026, linear mixed effects modeling, Cohen’s d = 0.65). Bayesian analysis provided anecdotal (weak) evidence in favor of the alternative hypothesis (BF_10_ = 2.6; see STAR Methods for details).

### Control analyses for non-perceptual task performance and variability

Longer phase durations in patients with structural lesions of the IFC may suggest that the IFC plays a contributing role for triggering changes in conscious experience during bistable perception. Alternatively, however, IFC lesions may impact non-perceptual functions such as task engagement, introspection, or motor behavior. However, we found no evidence for an effect of lesion location on the speed at which changes in conscious experience were reported (“response times: ”IFC 0.69 ± 0.18 s, non-IFC 0.70 ± 0.16 s, T(14.69) = 0.37, *p* = 0.72), the ability to perceive clear direction of rotation in ambiguous stimuli (“frequency of unclear perceptual states”: IFC 2.8 ± 16.3%, non-IFC 0.95 ± 8.2%, z = 0, *p* = 1), or the accuracy of perception during viewing of unambiguous control stimuli (“accuracy in the unambiguous control run”: IFC 88.33 ± 13.05%, non-IFC 85.0 ± 17.46%, z = −0.03, *p* = 0.97) (Figure 1C and Table 1). Further, post-hoc tests revealed no significant effect of age (T(11.01) = 0.68, *p* = 0.51), sex (T(11.38) = 0.03, *p* = 0.98), NIHSS at discharge (T(11.92) = −0.53, *p* = 0.61), and lesion volume (T(134) = −1.80, *p* = 0.09) on phase duration.

To determine whether the observed group differences in phase duration were due to increased variability of phase duration in the IFC group, we conducted a simulation analysis, which showed that the observed group difference (*p* = 0.026) was highly unlikely to be driven by variance alone (Figure S1B). Additionally, a Fligner-Killeen test of variability revealed no significant group difference in phase duration variance (*p* = 0.3). Furthermore, individual variability in phase durations was not significantly correlated with their average (r = 0.13, *p* = 0.6), supporting the conclusion that IFC lesions cause a true shift in the dynamics of perceptual updating rather than merely increasing variability. To further ensure that the observed group difference in phase duration (Figure 1C) was not dependent on the choice of mixed-effects modeling, we performed an additional one-sided permutation test, which showed a directional trend of longer phase durations in the IFC group (*p* = 0.072), supporting the robustness of the finding while warranting cautious interpretation.

## Discussion

Our results show that patients with ischemic stroke lesions in the right IFC exhibit significantly longer phase durations, and therefore fewer changes in conscious experience, than control patients with adjacent lesions not affecting the IFC. This finding provides rare lesion-based evidence that IFC plays a relevant, contributing role in the resolution of perceptual ambiguities, complementing prior TMS and neuroimaging studies that have been limited by confounds such as non-specific stimulation effects.^1^^,^^2^^,^^3^ Historically, reduced perceptual changes in visual ambiguity were already suggested in patients with frontal, parietal and occipital brain injuries.^13^^,^^14^ While these foundational studies lacked methodological precision in imaging as well as behavioral testing, they clearly anticipated the idea that distributed cortical networks underlie bistable perception.

IFC might regulate the access to awareness of sensory information that is in conflict with the currently dominant perceptual interpretation of an ambiguous stimulus.^1^^,^^10^ Recent functional magnetic resonance imaging (fMRI) studies have suggested that feature-selective regions in the visual cortex (such as V5/hMT+ for motion stimuli) detect conflicts between the contents of conscious experience and the underlying ambiguous sensory information during bistable perception.^7^^,^^10^ These conflict signals may be fed forward to a frontoparietal network, which is thought to temporarily reduce perceptual conflict by triggering a change in conscious experience.^7^^,^^10^^,^^15^ This view is motivated by hierarchical predictive processing, where prediction errors are thought to be fed forward from lower to higher levels of the cortical hierarchy to drive changes in conscious experience in response to conflicting sensory information.^16^^,^^17^ Indeed, work on the involvement of the parietal cortex in bistable perception has uncovered a functional subdivision, with posterior regions driving conflict-induced changes in conscious experience via prediction errors, and anterior regions stabilizing the interpretation of bistable stimuli.^18^^,^^19^^,^^20^ By extending this account to the prefrontal cortex, recent studies have shown that prediction errors propagate from the visual cortex to the IFC^7^ and that “virtual” IFC lesions induced by inhibitory repetitive transcranial magnetic stimulation (rTMS) reduce the rate of perceptual changes during bistable perception.^10^

While the functional representation of prediction errors in parietal and prefrontal cortex has been replicated multiple times,^1^^,^^10^ the causal evidence for an active role of the frontoparietal network in bistable perception has been limited in at least two ways. First, the use of rTMS over the IFC typically generates jaw muscle movement, which induces non-specific effects that may interfere with sensory processing and task engagement. Second, when applied over subregions of the parietal cortex, non-invasive brain stimulation has produced inconsistent effects on bistable perception.^19^ This variability raises questions about the reliability of non-invasive brain stimulation in pinpointing specific roles of cortical regions involved in the dynamic updating of conscious experience. This concern is particularly relevant for the evaluation of IFC. Here, studies using non-invasive stimulation have shown similarly diverging evidence for its effect on bistable perception.^18^^,^^20^ These inconsistencies further emphasize the challenge of drawing causal conclusions from stimulation studies.

By analyzing the effect of structural lesions to the IFC on bistable perception in the chronic lesion phase (≥6 months after symptom onset), we demonstrate that the IFC plays a relevant, contributing role in the resolution of perceptual ambiguity,^1^^,^^2^^,^^3^ while circumventing confounds related to the heterogeneity and unspecific acute effects of non-invasive brain stimulation. At the same time, we replicate the observation that the effects of IFC lesions are perceptual in nature and cannot be reduced to changes in the ability to engage with or reflect on the task of reporting changes in conscious experience during bistable perception, as previously suggested.^7^^,^^10^^,^^21^

Conversely, in an ongoing debate on the role of the frontal cortex in perceptual awareness, a multitude of work has implicated a predominant role of IFC in feedforward processing or downstream attentional mechanisms in response to bistable perception, rather than considering its role causal to perceptual change.^22^^,^^23^ A similar discourse, discerning the cause and effect of frontal cortex activity, has evolved regarding binocular rivalry.^8^^,^^24^ In both discussions, IFC’s involvement is considered primarily important for more general perceptual or motor mechanisms in perception. While we did not include a separate motor alternation control task (e.g., self-paced alternating button presses), our control analyses make it unlikely that a difference in perceptual change after IFC lesions rests solely on differences in attention. Accuracy in the unambiguous control condition and reaction times in both ambiguous and unambiguous runs did not differ between groups, indicating intact basic attention, engagement, and motor control. Moreover, we found no evidence for increased variability in alternation timing in the IFC group, which might be expected if a general self-paced switching mechanism were disrupted. Instead, the effect was characterized by consistently longer phase durations, aligning with a selective disruption in the resolution of perceptual ambiguity. Thereby, our work speaks to the central debate regarding frontal cortex activity in conscious perception.

Recent work by the Cogitate Consortium has sought to adjudicate this debate through preregistered, multisite studies comparing competing theories.^25^^,^^26^ Their results suggest that content-specific activity and connectivity in both sensory and frontal areas may be necessary under certain task demands, while frontal involvement may be dispensable when report requirements are minimized. The lesion evidence presented here adds causal weight to this view by showing that damage to the inferior frontal cortex significantly reduces the frequency of spontaneous changes in bistable perception, even when controlling for report-related confounds. This supports the notion that prefrontal structures, and the IFC in particular, may not merely reflect post-perceptual processes but play an active role in the dynamic updating of conscious content. Nonetheless, future work incorporating both perceptual and non-perceptual alternation tasks will be important to further clarify the functional specificity of IFC involvement.

Further, it should be noted that our findings do not refute the idea that IFC activity is implicated in feedforward processing in response to perceptual mechanisms occurring within sensory cortices.^4^^,^^5^ According to predictive processing accounts of bistable perception, a change from one percept to another is driven by prediction errors that indicate the conflict between the content of conscious experience, which reflects one interpretation of the external world, and sensory information that supports an opposing interpretation. While prediction errors may have an effect already at the level of the visual cortex, as evidenced by changes in conscious experience during bistable perception that occur without the involvement of parietal and prefrontal cortex,^4^ it has repeatedly been shown that these conflict signals are fed forward to the IFC.^7^^,^^10^ Our previous and current results confirm that the processing of prediction errors in the IFC is relevant for the resolution of perceptual conflict. Future studies will need to investigate whether this possibly causal role of the IFC is due to a representation of perceptual content in this region^8^^,^^9^ or due to feedback signaling to the visual cortex.^11^

This study has several limitations. Firstly, our sample size is small, limiting analytical approaches such as voxel-based lesion symptom mapping or connectome analyses. That said, our sample is comparable in size to similar studies evaluating cognitive processes in patients with stroke.^27^^,^^28^ Including only patients with lesions in one vascular territory and excluding lesions other than ischemic stroke is a strength of our sample. Our approach allows for a better comparability of groups and rules out possible secondary lesion effects such as edema in hemorrhagic lesions or tumors.^29^ However, given the modest sample size and moderate level of statistical support, our findings should be regarded as preliminary and would benefit from independent replication in larger cohorts.

We must acknowledge a certain heterogeneity regarding lesion size, with significant differences in lesion volume between groups. This is likely due to the fact that stroke lesions involving more peripheral areas of the MCA-territory, such as IFC, are typically either territorial or scattered lesions, and therefore tend to be larger.^30^ This was also true for our IFC sample. We observed a non-significant trend of shorter phase durations in individuals with larger lesions. However, the main effect of stroke location on phase durations — specifically, the longer phase durations observed in the IFC group — remained significant even when controlling for lesion volume. Our results suggest that lesion volume and IFC involvement possibly exert opposing effects on phase duration, with shorter phase durations for larger lesion volumes and longer phase durations for lesions specific to IFC. Stroke burden at discharge, which was higher in the IFC group as measured by NIHSS, showed a non-significant negative effect on phase duration, further supporting that the observed differences are primarily driven by IFC involvement.

None of the clinical parameters, including lesion volume, NIHSS at discharge, and age, affected our main finding when used as covariates in the statistical analyses. Non-perceptual task performance, which is likely to decline with age and larger stroke burden, did not differ between groups. This suggests that the effect of a longer phase time in the IFC-group relates to a perceptual function of IFC and not to an overall age-or stroke-related impairment. However, as we did not incorporate further neuropsychological testing, we cannot fully exclude the possibility of undetected differences in domain-general cognitive abilities such as memory or executive function. Our unambiguous control condition does not fully exclude contributions from higher-order executive or attentional mechanisms involved in endogenous switching. Future studies incorporating non-perceptual alternation tasks will be important to dissociate these potential influences.

It should also be noted that residual confounding of our results by overall lesion size or injury severity cannot be ruled out definitively. Because the lesions also affected adjacent frontal regions and underlying white-matter pathways, and because the sample size precluded voxel-wise lesion-symptom mapping, the present study cannot establish precise anatomical specificity. IFC involvement should therefore be viewed as the most parsimonious interpretation rather than a definitive localization.

Finally, due to the heterogeneity of lesion distribution, cortical areas beyond the frontal cortex may have affected the updating of conscious experience during bistable perception in our experiment. For example, the parietal cortex has been discussed as a relevant region in the resolution of perceptual conflict and is partially affected in our sample on the single-subject level. However, we saw no consistent overlap in lesion location at the group level in the inferior parietal sulcus and the temporoparietal junction in our sample, which have been considered most significant for conscious perception (Figure 1B).^1^^,^^20^ We therefore deem group differences in parietal lesioning as the primary driver of our effect unlikely. Further studies with larger sample sizes would be needed to differentiate between frontal and parietal involvement.

In conclusion, our results indicate that ischemic lesions in the IFC reduce perceptual changes during the viewing of bistable stimuli. In a tentative predictive-coding interpretation, this finding emphasizes the important role of IFC in the resolution of perceptual ambiguities and thus, in determining the contents of conscious perception. Understanding how our brains generate and update the contents of consciousness has significant implications for the neural foundations of consciousness. It may also clarify the mechanisms behind psychotic symptoms such as hallucinations, which have been linked to dysregulated access consciousness and disruptions in the updating of perception in response to conflicting sensory information.^26^

### Limitations of the study

The study is limited by a modest sample size, which constrained lesion-symptom mapping approaches and necessitates cautious interpretation of the findings. Residual confounding by lesion size, injury severity, or involvement of adjacent cortical and white-matter regions cannot be fully excluded, and the lack of comprehensive neuropsychological testing limits the assessment of domain-general cognitive contributions. These limitations are discussed in detail in the discussion and highlight the need for replication in larger cohorts.

## Resource availability

### Lead contact

Further information and requests for resources should be directed to and will be fulfilled by the lead contact, Merve Fritsch (merve.fritsch@charite.de).

### Materials availability

This study did not generate new unique reagents.

### Data and code availability

• All data reported in this article will be shared by the lead contact upon request.
• All original code generated in this study has been deposited at the Open Science Framework and is publicly available under the accession DOI https://doi.org/10.17605/OSF.IO/TGHMP, as listed in the key resources table, as of the date of publication.
• Any additional information required to reanalyze the data reported in this article is available from the lead contact upon request.

## Acknowledgments

V.W. and J.M. are fellows of the Clinician Scientist Program funded by the 10.13039/501100002839Charité – Universitätsmedizin Berlin and the 10.13039/501100017268Berlin Institute of Health. V.W. is further supported by the 10.13039/501100013368German National Academy of Sciences Leopoldina (grant number: LDPS2022-16). The authors thank Prof. Dr. Lukas Volz for helpful comments on an earlier version of the article.

## Author contributions

M.F., V.W., and P.S. conceptualized the study. J.S. and C.N. aided in conceptualization. V.W. designed the experiments. M.F., J.M., and L.J. collected the data. I.R., C.R., and C.N. aided in recruitment. M.F., V.W., and P.S. wrote the initial draft and edited the article. All authors reviewed the article.

## Declaration of interests

The authors declare no competing interests.

## Declaration of generative AI and AI-assisted technologies in the writing process

AI-assisted tools (ChatGPT, OpenAI) were used exclusively for language and grammar refinement; all scientific content, analyses, and interpretations were produced by the authors.

## STAR★Methods

### Key resources table

**Table:** 

REAGENT or RESOURCE	SOURCE	IDENTIFIER
**Software and algorithms**		

Psychophysics Toolbox 3	Brainard et al., 1997; Kleiner et al., 2007	RRID: SCR_002881

MATLAB R2019b	The MathWorks	RRID: SCR_001622

MRIcro	Chris Rorden	RRID: SCR_008264

SPM12	Wellcome Center for Human Neuroimaging	RRID: SCR_007037

SPM clinical toolbox	NITRC	RRID: SCR_014096
R Studio	Posit Software	RRID: SCR_000432
R (version 4.2.3)	R Foundation for Statistical Computing	RRID: SCR_001905
lme4 (R package)	CRAN	RRID: SCR_015654
Performance (R package)	CRAN	–
Brms (R package)	CRAN	RRID: SCR_023862
MRIcroGL	Chris Rorden	RRID: SCR_017779
Custom analysis code	This study	Database: OSF; DOI: https://doi.org/10.17605/OSF.IO/TGHM

### Experimental model and study participant details

Patients were recruited through the Stroke Unit of the Department of Neurology at Charité Campus Benjamin Franklin, Berlin, between 12/2019 and 12/2022. For comparison of patients with and without right IFC-lesions, we included only patients with acute right-hemispheric ischemic stroke within the right middle cerebral artery (MCA) territory. This ensured inclusion of IFC-lesioned patients in part of the sample and allowed a high comparability between groups.

Patients with acute lesions in other vascular territories as well as with chronic lesions in the posterior territory were excluded. Further exclusion criteria were severe or uncorrected visual impairment as well as pre-existing neurological or psychiatric comorbidities. Participants gave written, informed consent. All procedures were approved by the ethics committee at Charité – Universitätsmedizin Berlin (approval number: EA1/009/19). Overall, 46 patients were recruited. Out of the initially recruited patients, 16 patients could not be reached for a follow-up, or were not interested in further participation in our behavioral study. Further, 7 suffered recurrent stroke or another illness preventing further participation. Finally, 23 patients (11 female, mean age 70.65 ± 1.2 years) with chronic right-hemispheric lesions within the MCA-territory participated in our behavioral study and were included in our analyses. Stroke severity at admission and discharge was assessed using the National Institutes of Health Stroke Scale (NIHSS^31^). Behavioral data was collected in the chronic lesion phase (≥6 months post-stroke, mean time since stroke onset 16.2 months^32^) at the Department of Psychiatry at Charité Campus Mitte, Berlin (Table 1). Groups did not differ significantly regarding age (IFC 69 ± 9.6, non-IFC 71 ± 11.3 years, T(2.17) = 3.41, *p* = 0.07). There were significant between-group differences in stroke severity, as measured with the National Institutes of Health Stroke Scale (NIHSS),^31^ at discharge (IFC 2.0 ± 2.6, non-IFC 1.0 ± 1.3 points, T(177.66) = −6.03, *p* = 9.4 x 10^−9^), sex (female, IFC 44 ± 0.5%, non-IFC 46 ± 0.5%, T(4.77) = 0.09, *p* = 9.3 x 10^−1^) and lesion size (IFC 45154 ± 51694 mm^3^, non-IFC 903 ± 1137 mm^3^, T(181) = −9.27, *p* = 2 x 10^−16^). Data on gender identity, race, ethnicity, ancestry, and socioeconomic status were not collected, as these variables were not required to address the study’s research questions; this may limit the generalizability of the findings.

### Method details

#### Imaging

Participants received routine clinical stroke imaging, including diffusion-weighted imaging (DWI, repetition time = 2500 ms, echo time = 435 ms, slice thickness 2.5 mm) and fluid-attenuated inversion recovery (FLAIR) MRI sequences. Acute lesions were diagnosed in DWI sequences with 2.5 mm slice thickness. Patients later received additional imaging at the time of behavioral assessment, consisting of FLAIR and T1 sequences, to verify lesion distribution and control for recurrent stroke. Both imaging sessions were performed on 3-Tesla Siemens Magnetom Prisma scanners respectively (Siemens Medical Solutions, Erlangen, Germany). Allocation to “IFC” and “non-IFC” lesion groups was carried out by a neurologist blinded for behavioral and clinical data and not involved in data analyses using the Automated Anatomic Labeling (AAL) atlas.^33^ Lesions within the right IFC were defined as being located in the area comprised of the anterior insula and the inferior frontal gyrus (pars triangularis and pars opercularis). Nine patients showed MCA-lesions affecting the right IFC (“IFC”), and 14 patients showed MCA-lesions without affecting the right IFC (“non-IFC”) (an overlap of the lesions for the IFC and non-IFC group respectively is shown in Figure 1B).

#### Behavioral experiment

Patients performed a bistable, structure-from-motion task established in prior work of our group.^10^ Participants were asked to passively observe a spherical, discontinuous RDK and report changes in the perceived direction of rotation of the front surface (Figure 1A and Video S1). Perception of the spherical object was induced by the formation of random dots into two intersecting rings rotating around a vertical axis (diameter: 15.86°, rotational speed: 12 s per rotation, rotations per block: 10, individual dot size: 0.12°) (Figure 1A and Video S1). In the spherical RDK, changes in the perceived direction of rotation are most likely to occur when the two intersecting rings overlap, as this causes depth-symmetry which is needed for changes of subjective experience.^10^^,^^12^^,^^21^ In total, participants performed four runs, each consisting of six blocks (120 s each) that were separated by 10 s of fixation intervals. In the first run, a completely unambiguous version of the stimulus was presented to assess participants’ task performance. The stimulus was disambiguated by attaching a stereo disparity signal (1.8° visual angle) to all dots on the stimulus surface using dichoptic presentation with red-and-blue filter glasses that need to be worn throughout the run (left eye: red channel, right eye: blue channel) (Video S2). Here, direction of rotation changed with a probability of 15% per overlap (on average every 10 s) throughout the block by inverting the stereo disparity signal, thus creating stimulus-driven, exogenous changes in conscious experience. In all following runs, the sphere was presented without stereo disparity, thus holding equal amount of stimulus evidence for left- and rightward rotation. Participants were uninformed regarding the potential ambiguity of stimuli and were instructed to report changes in rotation direction via button-press on a keyboard, differentiating the rotation of the stimulus’ front-surface between (i) left, or (ii) right, or (iii) unclear direction. Stimuli were presented on an LCD-Monitor (60 Hz, 128031024 pixels, 60 cm viewing distance, 37.82 pixels per degree visual angle) using Psychophysics Toolbox 3 (RRID:SCR_002881) and MATLAB R2019b (RRID:SCR_001622).^34^^,^^35^

### Quantification and statistical analyses

#### Imaging analyses

Lesions were manually drawn on the DWI sequences using MRIcro (RRID:SCR_008264).^36^ DWIs and the corresponding, individual lesion maps were then re-oriented, normalized and resliced using SPM 12 and the Clinical Toolbox (RRID: SCR_007037 and SCR_014096),^35^^,^^37^^,^^38^ running on MATLAB (RRID:SCR_001622).^34^ A T1 template originating from elderly subjects and provided by the Clinical Toolbox was used for normalization.^39^ Further, lesion maps were registered to MNI standard space (Montreal Neurological Institute, MNI152 atlas, 1 × 1 × 1 mm isovoxels). Lesion overlay maps were created in MRIcroGL (RRID:SCR_017779).^36^ For the group-level overlay shown in Figure 1B, lesion maps were thresholded at 0.5 to enhance visualization of overlap; for individual lesion maps shown in Figure S2, no thresholding was applied to preserve full lesion extent.

#### Behavioral analyses

We measured bistable perception in terms of phase duration, i.e., the elapsed time between perceptual changes – as marked by button presses - in seconds. Phase duration was computed only for participants who reported at least one perceptual switch in the disambiguated runs. Two participants did not report any perceptual switches and were therefore excluded from the phase duration analyses (i.e., failure to elicit bistable perception). As proxies for non-perceptual task performance, we computed accuracy (the proportion of inter-overlap intervals where the reported experience matched the 3D cue is the disambiguated run 1), unclear perceptual states (the proportion of inter-overlap intervals where participants reported an unclear direction of rotation) and response times (the time difference in seconds between a reported change in conscious experience and the last overlapping configuration of the stimulus). The stimuli used in this experiment are discontinuous structure-from-motion stimuli. In these stimuli, perceptual changes occur almost exclusively when the object become depth-symmetric, i.e., when the rings that generate the illusory experience of an object rotating in depth overlap. We refer to these timepoints as overlaps (Figures 1A and S1A, Video S1).^10^^,^^12^

#### Statistical analyses

We conducted statistical analyses using MATLAB and R (version 4.2.3, RRID:SCR_001905).^34^^,^^40^ We fitted general linear mixed-effects models (GLMs) using the lme4 package (version 4.2.3, RRID:SCR_015564)^41^ to test the effects of group (categorical), age (continuous), gender (categorical), NIHSS at discharge (continuous), and lesion volume (continuous) on dependent variables, including phase duration, response times, fraction of unclear perceptual experiences, and perceptual accuracy. Models included fixed effects of interest (e.g., group) and random intercepts for age, gender, NIHSS at discharge, and lesion volume to account for individual variability. We used linear models for continuous outcomes (phase duration and response times) and binomial models with a logit link function for proportional outcomes (e.g., unclear perceptual experiences and perceptual accuracy). To explore the individual effects of age, gender, NIHSS at discharge, and lesion volume on phase duration, we conducted post-hoc control tests. Each control model contained the index predictor (e.g., age) as a fixed effect, with the other predictors (e.g., gender, NIHSS at discharge, and lesion volume) included as random intercepts nested within groups.

Marginal R^2^ was calculated to assess the variance explained by the fixed effects in the linear model. This value was obtained using the performancer2() function in R, which computes the marginal R^2^ based on the model fit.^42^ To validate our findings, we also conducted model-free analyses, including a one-sided permutation test and simulation-based checks for variance asymmetry. Cohen’s d was used to estimate the effect size between groups, calculated as the difference in means divided by the pooled standard deviation. Additionally, Bayes factors were computed to compare the relative evidence for each model, with a Bayes factor greater than 1 indicating stronger support for the full model over the alternative. The Bayes factor was calculated using the bayes_factor() function from the brms package.^43^ To control for over-specification of the model, we compared the full model to two reduced models with different random effects structures. Test Model 2 included random intercepts for age, NIHSS at discharge, and sex, while Test Model 3 only included random intercepts for age and sex. AIC and BIC values (Test Model 2: AIC 1392, BIC 1411; Test Model 3: AIC 1617, BIC 1634) were used to assess model fit, with the full model showing better performance (AIC 1294, BIC 1315). To test for relevant differences in variance of phase duration between groups, we used the Fligner-Killeen test to compare variances. Additionally, simulation analyses under the null hypothesis and a correlation analysis between variability and phase duration were performed to assess whether the observed group differences could be explained by increased variability.
