# Supplementary material for: Ischemic lesions to the inferior frontal cortex slow perceptual switching
Source: iScience. 2026 Feb 7;29(3):114943. doi: 10.1016/j.isci.2026.114943 (PMC12993236; doi:10.1016/j.isci.2026.114943)
Supplement: Document S1. Figures S1 and S2 [file mmc1.pdf]

iScience, Volume *29*

Supplemental information

Ischemic lesions to the inferior frontal cortex slow perceptual switching

Merve Fritsch, Jochen Michely, Lucca Jaeckel, Ida Rangus, Christoph Riegler, Jan F. Scheitz, Christian Nolte, Philipp Sterzer, and Veith Weilnhammer

Supplementary Figure 1 - Task structure and control analyses of phase duration variability.

A): Schematic Illustration of unambiguous (top) and ambiguous (bottom) runs: in the first run, a disambiguated version of the stimulus was presented to assess task performance. Here, direction of rotation changed every 10 seconds on average, thus creating stimulus-driven changes in conscious experience. In the following ambiguous runs, the sensory signal held equal stimulus information for left- and rightward rotation without changing over time. Here, spontaneous perceptual change is most likely to occur at the overlapping configuration of the stimulus, as this causes depth-symmetry. In both types of runs, patients reported perceptual changes via button press, allowing the calculation of reaction time (time between the button press and the last preceding overlap) as well as phase time (time between two indicated perceptual changes).

B): Simulation results testing the null hypothesis that the observed group difference in phase duration is due solely to increased variance in the IFC group (simulation with 10,000 datasets with equal group means and variances matching those observed). The observed p-value of 0.026 falls in the lowest 2.6% of this distribution.

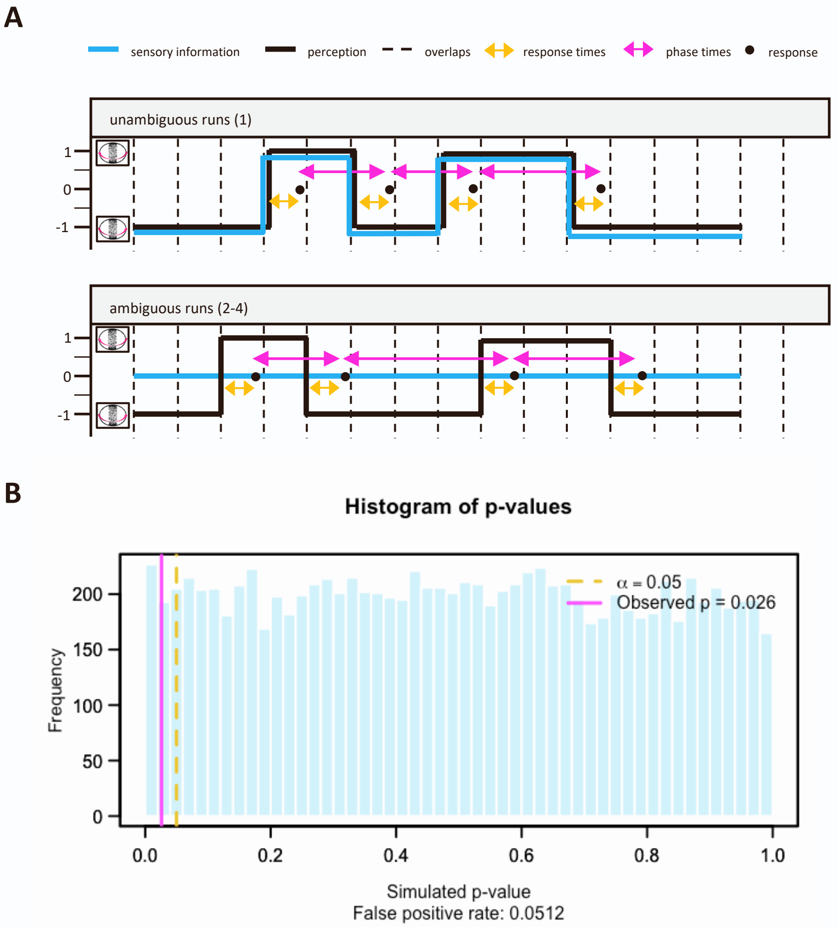
A
sensory information
perception
overlaps
response times
phase times
response
unambiguous runs (1)
1
0
-1
ambiguous runs (2-4)
1
0
-1
B
Histogram of p-values
Frequency
200
150
100
50
0
0.0
0.2
0.4
0.6
0.8
1.0
α = 0.05
Observed p = 0.026
Simulated p-value
False positive rate: 0.0512

Supplementary Figure 2 - Individual lesion maps of patients with and without right IFC involvement.

A) IFC group (lesions in red), subjects 3, 13, 14, 16, 17, 18, 20, 21, 23.

B) nIFC group (lesions in blue), subjects 1, 2, 4, 5, 6, 7, 8, 9, 10, 12, 15, 29, 24.

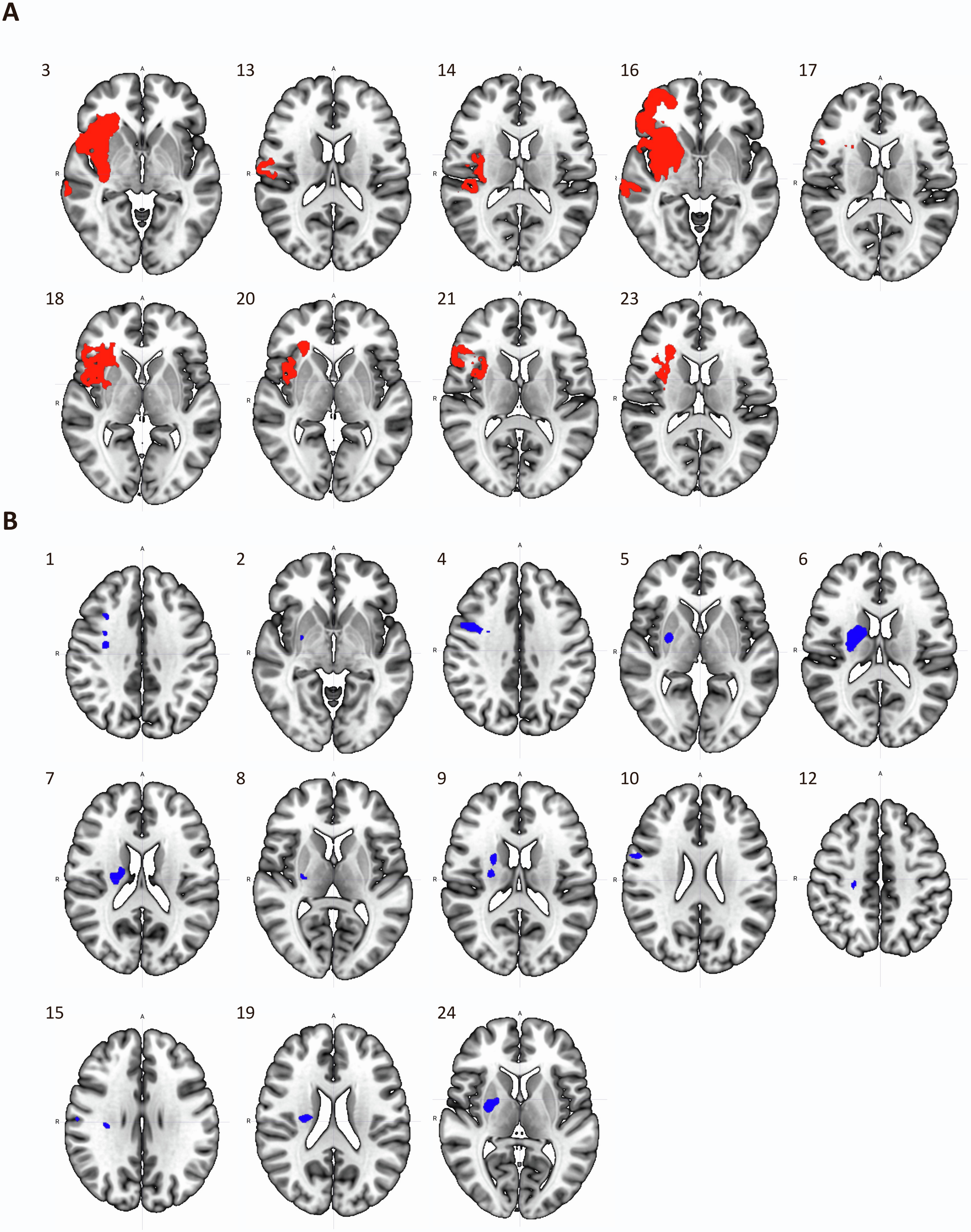
A
3
13
14
16
17
18
20
21
23
B
1
2
4
5
6
7
8
9
10
12
15
19
24

Supplementary Video S1 - Ambiguous structure-from-motion stimulus inducing bistable perception.

Ambiguous structure from motion, related to Figure 1A.

Supplementary Video S2 - Disambiguated structure-from-motion stimulus used in the control condition.

Video S2: Disambiguated version of the stimulus (during the experiment viewed with red-and-blue filter glasses).
